# American Health Primers

**Published:** 1879-07

**Authors:** 


AMERICAN HEALTH PRIMERS. Edited by W. W. Keen, M.D., Fellow of the College of Physicians of Philadelphia. Lindsay & Blakiston, Philadelphia—1879.

This publication is composed of a series of small volumes on subjects pertaining to Sanitary Science, and the perservation of health, written by American authors of established reputation, selected with reference to their special knowledge of the subject from previous study or as private and public teachers. They are written from an American standpoint, with particular reference to our climate and modes of life. The subjects selected are of vital and practical importance, and are treated in as popular a style as is consistent with their nature—technicalitiies of language being avoided. Each volume will be illustrated by engravings, when the text can thus be more fully explained to those not heretofore familiar with the structure or function of the body. They will be printed on toned paper, handsomely bound in cloth, and mailed, postpaid, upon receipt of price—fifty cents each.

